# Diffusion Behavior of VOC Molecules in Polyvinyl Chloride Investigated by Molecular Dynamics Simulation

**DOI:** 10.3390/ijerph20043235

**Published:** 2023-02-12

**Authors:** Yun-Feng Mao, Shun-Nan Long, Zhuo Li, Wen-Quan Tao

**Affiliations:** 1 School of Energy and Power Engineering, University of Shanghai for Science and Technology, Shanghai 200093, China; 2 State Key Laboratory of Pollution Control and Resource Reuse, Tongji University, Shanghai 200092, China; 3 Key Laboratory of Thermo-Fluid Science and Engineering of MOE, School of Energy and Power Engineering, Xi’an Jiaotong University, Xi’an 710049, China

**Keywords:** VOC, diffusion coefficients, molecular dynamics simulation, polymer

## Abstract

Due to the threats posed by many volatile organic compounds (VOCs) to human health in indoor spaces via air, the mass transfer characteristics of VOCs are of critical importance to the study of their mechanism and control. As a significant part of the mass transfer process, diffusion widely exists in emissions from floors (e.g., PVC floors) and in sorption in porous materials. Molecular simulation studies by can provide unparalleled insights into the molecular mechanisms of VOCs. We construct the detailed atomistic structures of PVC blend membranes to investigate the diffusion behavior of VOC molecules (n-hexane) in PVC by molecular dynamics (MD). The variation in the diffusion coefficient of n-hexane in PVC with respect to temperature is in line with Arrhenius’ law. The effect of temperature on the diffusion mechanism was investigated from the perspectives of free volume, cavity distribution and polymer chain mobility. It was found that the relationships between the diffusion coefficients of n-hexane in the polymer and the inverse fractional free volume are exponential and agree well with the free volume theory. Hopefully, this study will offer quantitative insights into the mass transport phenomena of VOCs within polymeric materials.

## 1. Introduction

Volatile organic compounds (VOCs) are a group of compounds widely distributed in indoor environments and can be easily found in various types of sources such as polyvinylchloride (PVC) plastic products with plasticizers, house furnishings with fire retardants, and personal care products [1]. These materials significantly contribute to indoor air pollution during the first six months after houses are furnished. This kind of indoor pollution persists for more than one year and a continuous, steady concentration higher than the outdoor concentration has been measured [2]. As widely used decorating materials and artificial product constituents, polymeric materials are important contributors of VOCs to the indoor air and pose a persistent health risk to humans. Taking PVC polymeric materials as an example, it is reported that VOCs are emitted at a rate as high as 120 µg/m^2^/h from carpets with PVC fibers, and at a rate of 22,280 µg/m^2^/h from PVC flooring [3]. The mass transfer process of VOCs from polymeric materials in the indoor environment is therefore a significant issue affecting indoor air quality.

A great number of VOCs can be emitted from indoor building materials, and some of them can be adsorbed on indoor surfaces [4]. Diffusion is involved in both the emission and sorption processes. The material-phase diffusion coefficient (*D*) is a key parameter that influences the mass transfer characteristics of emission from furnishings [4] and also influences those of adsorption in porous materials in an indoor environment [5]. However, the mechanism of diffusion in the mass transfer model of VOCs has not been deeply studied [6]. For example, the influences of ambient temperature and humidity on the VOC diffusion coefficient in building materials are ignored without any explanations. Due to technical difficulties in experiments, it is difficult to understand the diffusion mechanism through conventional experiments. Cox et al. (2001) [7] used a microbalance test system to measure the diffusion coefficient of VOCs, though it cost them several hundred hours to complete a full experiment. They only compared the diffusion coefficients of different VOCs, but no theoretical explanations on a diffusion mechanism were given. Therefore, a new method is urgently needed to investigate the diffusion mechanism.

Molecular simulation can provide a general method to explore various natural processes at the molecular level [8] based on the assumptions that the motions of atoms follow Newton’s first law and interactions among atoms are described by empirical potential functions. In principle, molecular simulation can provide unparalleled insights into the molecular mechanisms that govern the emission and sorption processes, often in ways that experiments cannot emulate [9]. It represents an interface between laboratory experiments and theories, which can be understood as a “virtual experiment” [10]. A few studies have applied molecular dynamics (MD) simulations in studying diffusion behaviors [11,12,13]. Kucukpinar and Doruker [14] calculated the diffusion and solubility coefficients of penetrants in nitrile rubber (NBR) and styrene butadiene rubber (SBR). They found that small gas diffusion in the polar matrix of NBR was significantly lower than that of SBR. Müller-Plathe [15] investigated the diffusion of water in poly(vinyl alcohol) (PVA) over a wide range of compositions using MD simulations, and found that the pattern of water diffusion changed pronouncedly with temperature. The hopping mechanism was applied to analyze this diffusion behavior. Noorjahan and Choi [16] also studied the diffusions of water and benzene in PVA, and they further investigated the effect of free volume on the diffusion of free volume redistribution. Calculations of free volume and its distribution were used to explain the mechanism of diffusion, and the calculated free volume results were compared with the results from positron annihilation experiments [17].

To our knowledge, no atomistic-level study on the diffusion of VOCs in PVC, present in typical indoor materials such as vinyl flooring, has been reported. N-hexane was chosen as a kind of VOC due to it posing less harm to human beings, so it is convenient to test its physical properties (such as its diffusion coefficient) in PVC materials. Hexane can be easily found in rubber flooring and nylon carpets and emits at a rate of ~3.5 mg/m^3^ from these materials [18]. The objective of this paper is to study the diffusion process of n-hexane in PVC at different temperatures by explaining the mechanism in terms of penetrant movement, PVC chain mobility, and the free volume.

## 2. Molecular Modelling

MD simulations were carried out using the discover and amorphous cell module of the Materials Studio package of Accelrys and the COMPASS force field (condensed-phase optimized molecular potentials for atomistic simulation studies).

### 2.1. Force Field

The COMPASS force field [19] was employed in this study. The force field was parameterized and validated using condensed-phase properties, including various ab initio and empirical data for molecules in isolation. The COMPASS force field has been successfully used for a long time in predicting various properties of the materials in the references [20,21,22]. It is a general force field for molecular dynamics simulation of common organic molecules, inorganic small molecules, and polymers such as polyethylene (PE) and polyvinyl alcohol (PVA). All the interaction potentials in the study were computed based on the COMPASS force field. The functional forms used in this force are the same as those used in CFF-type fields [23], which are described as follows [19]:

(1) $$E_{total}=E_{b}+E_{\theta }+E_{\phi }+E_{\chi }+E_{bb^{\prime }}+E_{b\theta }+E_{b\phi }+E_{\theta \theta^{\prime }}+E_{\theta \theta^{\prime }\phi }+E_{elec}+E_{LJ}$$

where *E_total_* represents the total energy function in the COMPASS force field, which can be decomposed into 12 valence and non-bonded interaction terms. Specifically, they are the bonding stretch *E_b_*, bending *E_θ_*, torsion *E_ϕ_*, and out-of-plane *E_χ_* potentials. In addition, *E_bb′_*, *E_bθ_*, *E_bϕ_*, *E_θθ′_* and *E_θθ′ϕ_* are off-diagonal cross-coupling terms used for describing the interactions between diagonal terms. *E_LJ_* and *E_elec_* are the short-range van der Waal and the long-range electrostatic interactions described by Lennard-Jones (L-J) 9-6 and Coulombic functions, respectively.

### 2.2. Generation of the Polymer Structure

The PVC chain was constructed with polyvinyl monomers directly utilizing the Polymerizer and Amorphous cell modules of MS 7.0. In this work, one polymer chain consisting of 200 repeat units was employed in the simulation cell so as to minimize the chain ends effect. The periodic boundary condition was used to eliminate the boundary effect. A Maxwell–Boltzmann distribution at the target temperature was used to assign the initial velocity of the polymer atoms, and then the cell was equilibrated to obtain a more realistic configuration. To make sure the simulated system was well equilibrated before the production, the generated cells were subsequently optimized by various steps. The energy of each constructed cell was minimized to a convergence value of 0.01 (kcal/mol)/Å. In order to well relax the system to a local state of minimal potential energy, two different methods were used. Firstly, the method of steepest descent was used to accelerate the calculation, and then the method of conjugate gradient further minimized the potential energy. After minimization, the conformation with the lowest energy was chosen. To prevent the system from being trapped at a local high energy, the cells were heated by 25 K from 300 K to 600 K, which is well above the glass transition temperature. Subsequently, 1 ns of NVT-MD simulation was performed at 600 K, and then cooled back to the desired temperature at 25 K increments. The timestep is 1 fs in the whole work. The density as a volumetric quantity was compared with the experimental data. In order to obtain the density, several NPT cycles were performed by increasing the pressure. In addition, NVT simulations were also performed at high temperature to relax the polymer chain and then the cell was cooled down to the target temperature. Finally, 10 ns NVT-MD dynamic simulations were run at the target temperature.

### 2.3. Diffusion in the Polymer

**Figure 1:**
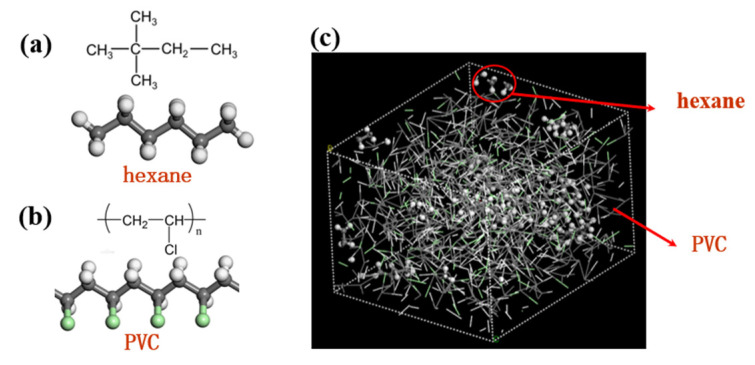
(**a**) n-hexane molecule; (**b**) PVC monomer; (**c**) snapshot of the simulation box.

A total of 10 penetrant molecules were randomly inserted at the free volume sites of the cells to avoid interatomic overlapping. The conformation with penetrant molecules separated from each other was chosen to disperse the molecules in the unit cell. Then, the system (the polymer plus the penetrant molecules) was relaxed through a series of 50 ns NVT and NPT runs at the target temperature and 1 bar of pressure before using them for data production. After half of the runs, the equilibrium density and energy for the desired temperature and pressure were judged to be attained by observing the energy fluctuating around a mean value along with the running time. At the end, the system was found to be in the most probable configuration (Figure 1).

### 2.4. Pore Size Distribution

The pore size distribution (PSD) provides a means of quantifying the distribution of sizes. A fast method developed by Battacharya and Gubbins [24] was used for computing pore size distribution. In brief, the simulation box in this work was divided into three-dimensional (3-D) fine grids with a size of approximately 0.1 Å. Some points were selected to be randomly and uniformly distributed in the simulation box. The void size at each of the selected points is determined as the size of the maximum cavity that could enclose the given point, without additional atoms overlapping with any polymer atom. The SOLVOPT program [25] was used to optimize non-smooth objective functions to obtain the maximum cavity. A cumulative histogram *H*(*D*) is obtained, which represents the probability of finding a point in the model space with a pore diameter greater than or equal to *D*. The pore size distribution *P*(*D*) is defined as [25]:

(2) P(D)=−dH(D)/dD

## 3. Results and Discussion

### 3.1. Diffusion Coefficients

The interest of this work is to analyze diffusion coefficients of VOCs in PVC and the diffusion mechanism contributed by both the PVC free volume and the sizes of the penetrants. A convenient and widely accepted way to validate the packing models obtained is to compare the predicted data with the experimental results. According to the kinetic uptake curves [26,27], the diffusion coefficient of n-hexane in the PVC sample can be estimated based on the uptake amount. However, the uptake amounts at high temperatures are too small to accurately determine the diffusion coefficients. So, in this work we validate the packing model by comparing our simulated PVC density with the data by Sacristan and Mijangos [28], as summarized in Table 1. From Table 1, the predicted PVC densities are very close to the experimental data offered by Sacristan and Mijangos, and both follow the same trend in that the PVC densities decrease slightly with the rising temperature.

**Figure 2:**
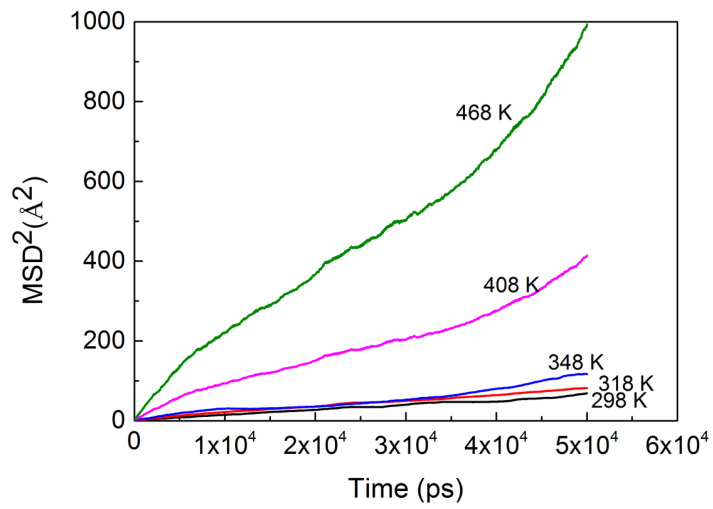
MSDs of n-hexane diffusion in PVC change with time at different temperatures.

As for diffusivity, the mean square displacement (MSD) obtained from the simulations was used in analyzing the diffusion coefficients with the Einstein equation [15]:

(3) $$D=\frac{1}{6}\underset{t\to \infty }{\lim}\frac{d}{dt}\sum_{i=1}^{N_{a}}\langle {\left(r_{i}\left(t\right)-r_{i}\left(0\right)\right)}^{2}\rangle$$

where <(*r_i_*(*t*)-*r_i_*(0))^2^> and *D* are the MSD of the penetrant and the diffusion coefficient, respectively. The results for the mean square displacement (MSD) of the penetrant molecules were calculated over a wide range of temperatures, from 298 to 468 K. To smooth the curves, averaging of MSDs was conducted over all the n-hexane molecules. The linear parts of the MSD curve in Figure 2 were least-squares fitted to calculate the diffusion coefficient.

Figure 2 demonstrates that a time scale in nanoseconds (ns) is long enough for this system to calculate the diffusion coefficient. From the center-of-mass mean square displacement of n-hexane at 298 K (in black color), we found that the n-hexane molecules are fairly localized, which could be attributed to the absence of large voids in the PVC or to the strong interactions between n-hexane molecules and the PVC chains, which are stronger than those between the n-hexane molecules themselves. Both reasons could cause the n-hexane to have difficulty in moving from site to site within PVC cavities. The rest of the lines are the center-of-mass mean square displacements of n-hexane in PVC at other temperatures, which are used to calculate the diffusion coefficients. From the slope of the lines, we could qualitatively compare the diffusion coefficients of different temperatures and found that the diffusion coefficients increase with temperature.

### 3.2. Temperature Dependence of the Diffusion Coefficients

As early as in 1937, Barrer [31] showed that the diffusion of molecules in rubbery polymers was a thermally activated process, and pointed out that diffusion in the polymer obeyed Arrhenius’ law:

(4) $$D=D_{0}e^{-\frac{E_{d}}{Rt}}$$

where *E_d_* is the apparent activation energy, *T* is the temperature and *R* is the gas constant.

**Figure 3:**
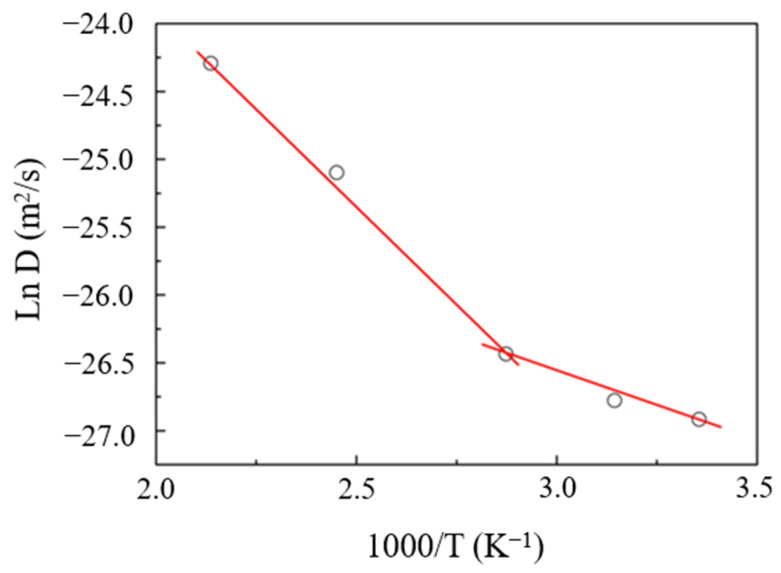
The log diffusion coefficient vs temperature for n-hexane calculated from MD simulations.

Various data in the literature suggest that the transport coefficients (namely permeation, diffusion and solubility) depend on temperature for a given pressure, via Arrhenius’ law [32]. Some researchers [33,34] studied self-diffusion coefficients by MD simulation, and found that the self-diffusion coefficients were a function of temperature via Arrhenius’ law. In this work, we used Arrhenius’ law to figure out the relationship between the diffusion coefficients and temperature for n-hexane. The results are diagramed in Figure 3, which indicates the log diffusion coefficient vs reciprocal temperature for n-hexane in PVC.

Figure 3 clearly shows that penetrant diffusivities are strongly dependent on temperature and increase with temperature. A slower penetrant mobility occurs at a lower temperature (298 K) compared with the mobility at a higher temperature (468 K). At low temperature, the penetrant spends more time trapped in a cage which is formed by the surrounding polymer chains, while at higher temperatures the jumps between neighboring sites become easier and more frequent. Compared with some studies on the diffusion of gases such as N_2_, CO_2_ and CH_4_ in polymers [35,36], n-hexane molecules have a larger size, leading to a more difficult diffusion in materials. Sacristanand Mijangos [30] noted that for the diffusion behaviors of larger molecules, larger cavities were required in the polymers. Therefore, greater energy for the formation of larger cavities is needed. In addition, a large activation energy is also required for the diffusion of larger molecules. Further examination of the plot in Figure 3 reveals a similarly linear relationship below the glass transition temperature (*T_g_*), with an abrupt, slight change in slope at the *T_g_* of PVC (354 K). From that we can deduce that the activation energy for diffusion is lower below *T_g_* than it is above. At temperatures above *T_g_*, the activation energy was calculated to be 24 kJ/mol, while below *T_g_* it was 8 kJ/mol. These different activation energies suggest that the mechanism for n-hexane diffusion in PVC changes above and below *T_g_*. This difference actually results from the change in free volume in PVC and the mobility of PVC chains caused by temperature variation. Further explanations regarding these aspects are presented in the following sections.

### 3.3. Analysis of the Diffusion Mechanism

#### 3.3.1. Mobility in PVC

**Figure 4:**
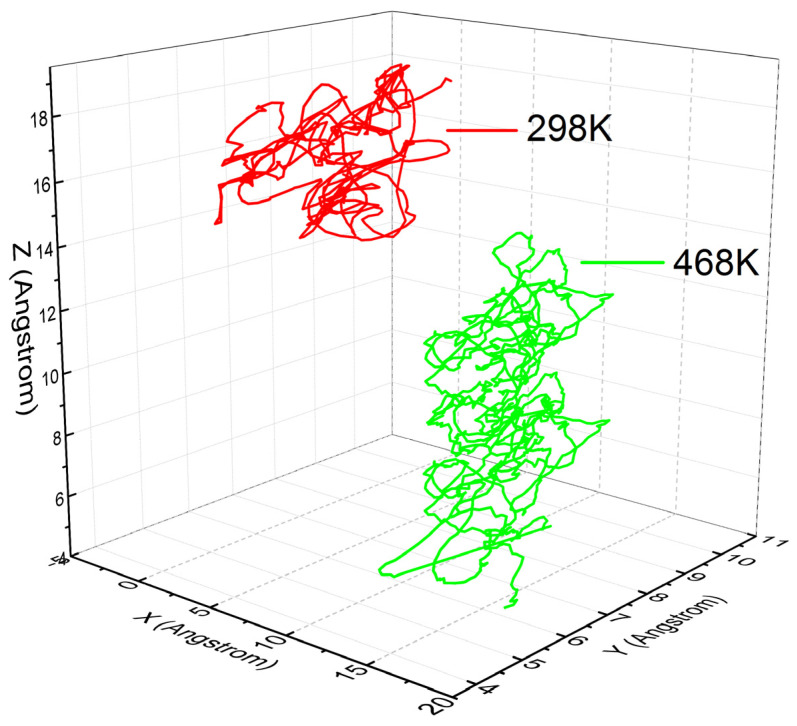
Part of trajectories of n-hexane molecules in PVC at 298 K and 468 K, respectively.

**Figure 5:**
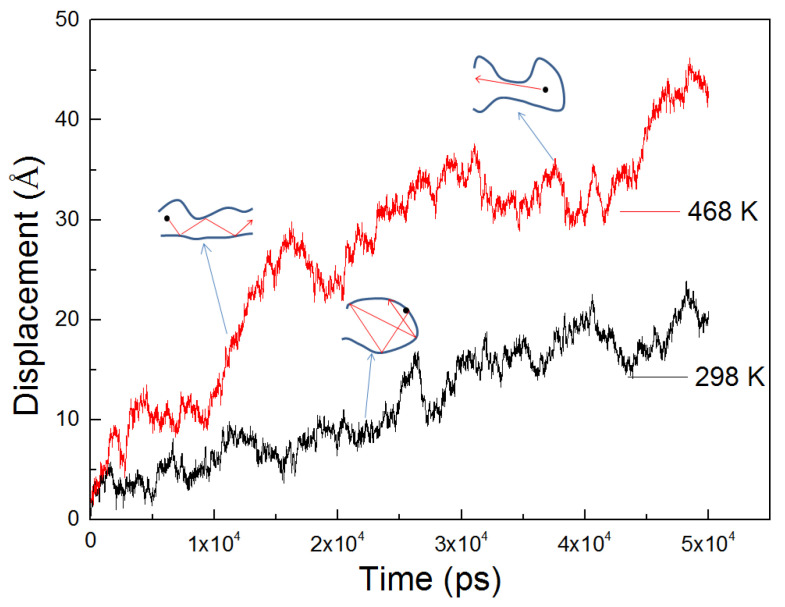
Displacement of center of mass of n-hexane in PVC at 298 K and 468 K, respectively.

The displacements of an n-hexane molecule in the first 1000 ps were chosen to qualitatively reveal the diffusion behavior of n-hexane in the PVC. As illustrated in Figure 4 and Figure 5, three types of molecule movements in the PVC can be clearly identified [32]: firstly, the rise of the curve in the beginning indicates diffusion through channels; secondly, the intermediate segment represents the penetrant jumping between polymer voids; and the third segment represents the n-hexane being stuck in the polymer void. Note that the back “jump” exists, but only the forth “jump” does favor to the diffusion process [33]. These three types of movements are hard to obtain by experimental methods. To investigate and compare the movements of penetrants at different temperatures, we present part of the trajectories of n-hexane molecules in PVC at 298 K and 468 K in Figure 4, and the total displacement |*r*(*t*)*-r*(0)| of the center of mass of n-hexane molecules in PVC at 298 K and 468 K in Figure 5.

In Figure 4, a wider movement of penetrants can be found at *T* = 468 K than at *T* = 298 K, which indicates that at higher temperatures the free volume of the holes in PVC can be redistributed to make it easier for penetrant molecules to overcome the activation energy that is required to jump into new voids. There exist three kinds of movements of n-hexane molecules in PVC, as labeled in Figure 5. N-hexane molecules frequently jump back and forth between two neighboring holes. This may be due to the existence of temporary channels between different parts of the free volume that are short-lived with respect to the time that the penetrant molecules spend inside the cavity [37,38]. Penetrant molecules also dwell in the voids by performing oscillatory motions around their equilibrium positions, which does not contribute to the net motion, and hence does not contribute to diffusion. The amplitude of oscillations depends on the size of holes, and a larger increase in amplitude indicates a jump between two adjacent holes. N-hexane molecules oscillate in the voids and try to escape from these voids in an easy configuration. These molecules will succeed in escaping if the PVC chain moves to create a larger channel between holes, and if penetrant molecules can avoid the interruption from the PVC side chain.

#### 3.3.2. Free Volume

Several factors determine the diffusion of penetrants in polymers, including temperature, chain rigidity and free volume [28]. Among them, free volume, as well as its distribution and topology, has been deemed to show complicated influences on the diffusion behavior of penetrants [39,40]. However, current experimental techniques usually fail to provide details of the structure of the free volume voids. Instead, molecular simulations allow researchers to understand the diffusion of the desired penetrants at small costs with high precision by providing the time evolution of a target system [34]. In this work, fractional free volume (*FFV*) is chosen to evaluate the polymer void structure, which is defined as the fraction of the volume not occupied by the polymer atoms. *FFV* can be calculated from the following empirical equations [39]:

(5) $$FFV=\frac{V-V_{0}}{V}$$

(6) $$V_{0}=1.3V_{w}$$

where *V* is the cell volume, and *V_w_* is the van der Waals volume. Due to the fact that *V* and *V*_0_ depend on temperature for a given sample, *FFV* likewise varies with temperature, according to Equation (5).

The *FFVs* at different temperatures were calculated according to Equations (5) and (6). Table 2 lists the *FFV* values from our simulation results, and the data from references [28]. From Table 2, *FFV* strongly increases with temperature, which is consistent with the trend of the other data. Apparently, the dependence of *FFV* on temperature is consistent with that of the diffusion coefficients of n-hexane molecules. At 298 K, where n-hexane molecules diffuse relatively slowly in the PVC polymer compared to the other studied temperatures, *FFV* is only 0.15, less than half of that at 468 K. The specific calculated *FFV* values are somewhat larger than those from Sacristan and Migangos [28] at 298 K, but the values are very close, while the difference becomes larger at higher temperatures. Another study [41] which analyzed the free volume of PVC by NPT Monte Carlo simulations, obtained even larger *FFVs* (0.3 at 373 K and 0.4 at 456 K). The reason could be the differences in polymer packing efficiency and density, respectively, between two simulations. Despite this, the calculated diffusion coefficients in the two simulations follow the same tendency with respect to temperature, and they are within a reasonable range.

**Figure 6:**
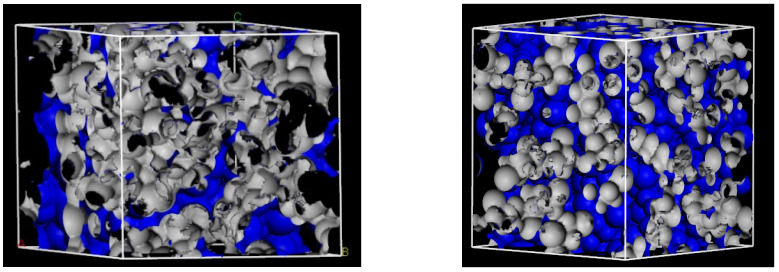
Three-dimensional representation of fractional free volume in PVC at 298 K (**left**) and 468 K (**right**).

To demonstrate the free volume distribution and its influences on the diffusion behavior, the morphologies of the *FFV* for PVC at 298 K and 468 K are displayed in Figure 6. From Figure 6, we can clearly see the change of the free volume distribution with temperature. A high temperature would lead to a compact structure, providing less empty volume available for penetrant transport. At higher temperatures, some small and dispersed free volumes become more continuous. 

**Figure 7:**
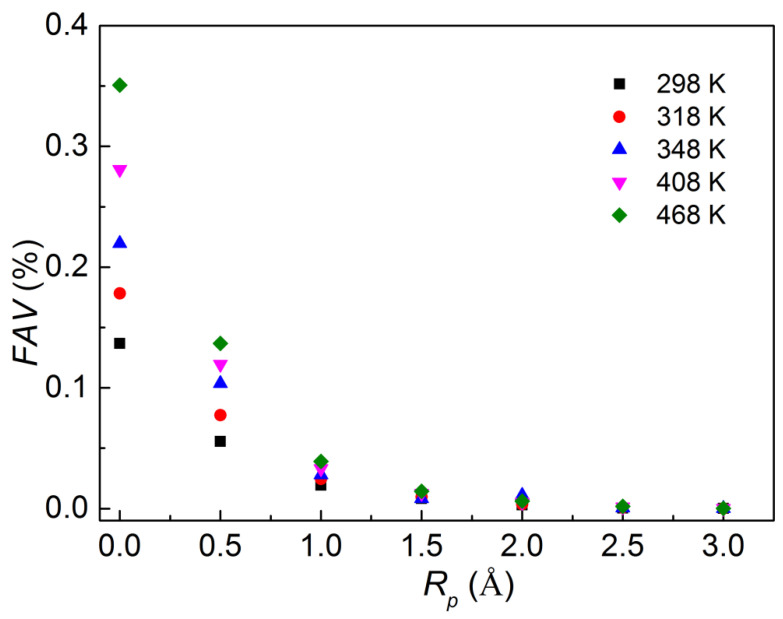
Fractional accessible volume calculated from MD simulations at different temperatures.

However, the *FFV* only gives the bulk property of free space in the polymer. The *FFV* includes some “dead” volume that is not accessible to the penetrant molecules, and the “dead” volume does not make a considerable contribution to the transport property of the polymer. Instead, the fraction accessible free volume (*FAV*) is defined as the amount of free volume that is accessible to the penetrant molecules relative to the total volume of the model. *FAV* was obtained using various sizes of the (spherical) hard probe particle to probe the available free volume of the polymer for a particle passing through with respect to the size of the penetrant molecule. The accessible volume of the polymer can be obtained from the Visualizer Module of the Materials Studio software. Hence, the *FAV* excludes the volume that is inaccessible to the probe. The dependence of *FAV* on the probe radius (*R_p_*) is shown in Figure 7.

Figure 7 clearly shows that the accessible volume in the PVC matrix follows the same trend as the *FFV*. However, a higher percentage of *FAV* is probed with a smaller probe radius, which indicates that the polymer matrix limits the movement of larger molecules within it. Temperature strongly affects the *FAV*, especially at smaller probe radii. Nevertheless, the influence decreases as the probe radius increases. This indicated that the temperature could not significantly improve the *FAV* for a larger probe radius, and as such it could not improve the diffusion of large molecules.

**Figure 8:**
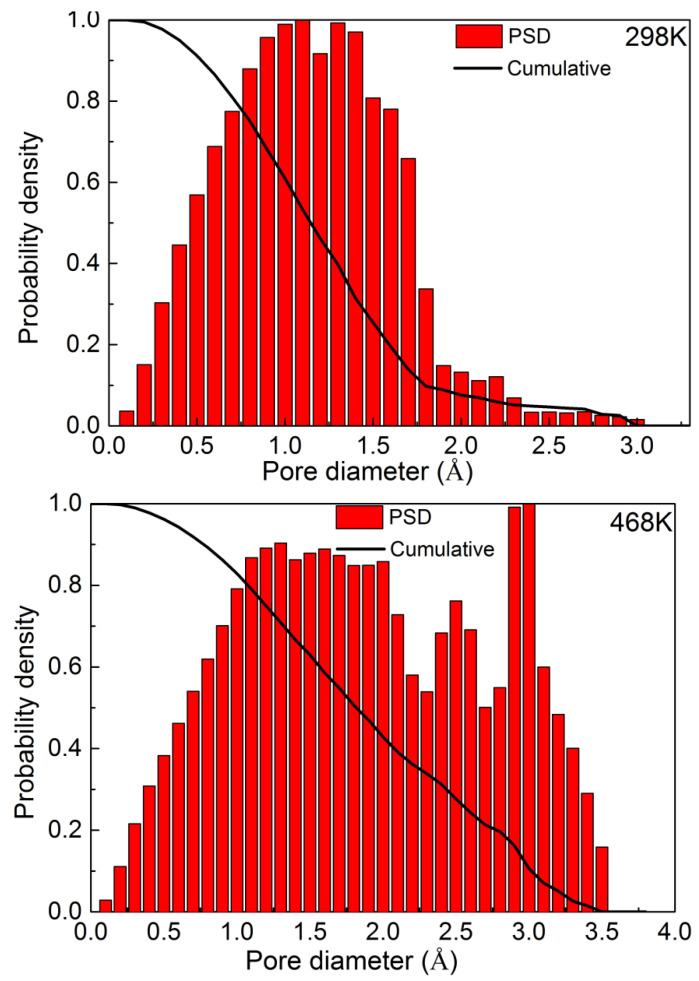
Pore size distributions in the PVC structure at 298 K (**top**) and 468 K (**bottom**).

Furthermore, some other factors beyond the overall free volume accessible to molecules should be taken into account [28], and it has been suggested that the topology of free volume should be discussed. Chang et al. [42] pointed out that in addition to the free volume size, the volume shape also controls the species transport, and it might dominate the transport mechanism. Figure 8 gives the pore size distributions for the model PVC structure at 298 K and 468 K, in which the PVC polymer exhibits a moderate range of void size distributions. Comparing the pore size distributions at different temperatures, we found that there is no apparent change in small voids, but the probability density of larger void spaces increases with the rise in temperature. The larger voids contribute predominantly to penetrant diffusion, and as we can see in Figure 2, the diffusion coefficient at 468 K is greater than that at 298 K.

#### 3.3.3. Local Dynamics of Polymer Chains

**Figure 9:**
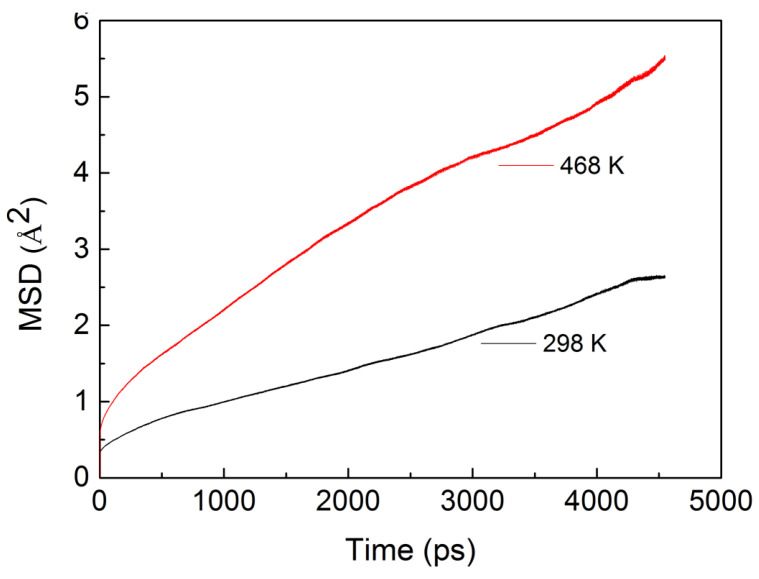
MSD vs time of polymer backbone carbon atoms for PVC.

Pant and Boyd [43] pointed out that polymer chain mobility plays a role in penetrant diffusion. Kucukpinar and Doruker [44] also indicated that the relaxation of polymer chains has great influence on penetrant diffusion in polymers. In order to investigate the role played by polymer chain relationships, the local dynamics of the PVC matrices at different temperatures are compared here by calculating the MSDs of the polymer backbone carbon atoms, as shown in Figure 9. This figure demonstrates that the slower motion of backbone atoms could well explain the slower diffusion in this polymer at lower temperatures, as obtained in Figure 1, and the higher temperature leads to faster motion of the backbone atoms. When the polymer is more mobile, the penetrant is less “trapped” in voids. The motion of backbone atoms hence creates some additional free volume available for the diffusion of n-hexane molecules. More free volume in a polymer could give increased segmental mobility, and result in a reduction in the energy that is required to overcome the interaction between adjacent polymer chains for penetrant molecule diffusion. Actually, the cooperative motion of a great many consecutive chain segments is easier above *T_g_*, which results in a greater activation energy (*E*). The greater temperature sensitivity of *D* above the *T_g_* might be thought of in terms of a greater thermal expansion coefficient of the fractional free volume. The fractional free volume in Table 2 increases mildly with temperature at temperatures lower than *T_g_*, and only localized in-chain motions are active. Therefore, we can conclude that the expansion of available free volume with temperature above the *T_g_* leads to the change in slope in Figure 3.

## 4. Conclusions

We constructed the detailed atomistic structures of PVC blend membranes to investigate the diffusion behavior of VOC molecules (n-hexane) in it. Molecular dynamic simulations were used to study diffusion in PVC in the temperature range of 298–468 K. The numerical results were compared with the results for PVC densities at different temperatures from references, with a good agreement achieved. The effect of temperature on the diffusion mechanism was investigated in detail from the perspectives of free volume, cavity distribution and polymer chain mobility. The simulated results give a detailed determination of molecular diffusion and structural parameters such as fractional free volume, fractional accessible volume and the distribution of fractional free volume, all of which are difficult to obtain by experimental methods. These results highlight the validity of MD simulations in calculating penetrant transport properties in PVC materials.

The chain packing structure of PVC at high temperatures becomes less structured than that at low temperatures. Consequently, the diffusion coefficient of n-hexane changes with temperature. In addition to accelerating the motion of penetrant molecules, temperature affects the diffusion coefficients by changing the free volume, distributions of free volume regions and pore size. High temperature increases the free volume as well as the probability of large pore size. In addition, high temperature could change some small and dispersed free volume regions into partly continuous free volume. The faster mobility of the backbone atoms, which resulted from higher temperatures, is also responsible for the higher diffusion coefficients calculated in this work. All of the above factors facilitate the penetrants’ escape from the trapped voids. Studies of diffusion mechanisms, which are a main part of VOC emissions from and sorption in materials, will help in understanding and controlling VOC pollution in indoor environments.

## Figures and Tables

**Table 1:** Experimental and calculated densities of PVC at different temperatures.

Van Krevelen and TeNijenhuis [29] K. Bierbrauer, et al. [30]	300 K	373 K	393 K		
	1.387	1.352	1.334		
Sacristan and Migangos [28]	300 K	375 K	400 K	425 K	450 K
	1.382	1.328	1.301	1.277	1.258
Calculated results	298 K	318 K	348 K	408 K	468 K
	1.401	1.387	1.338	1.307	1.273

**Table 2:** Fractional free volume in PVC as a function of temperature.

*FFV* [28]				
300 K	375 K	400 K	425 K	450 K
0.12	0.15	0.18	0.19	0.21
*FFV*				
298 K	318 K	348 K	408 K	468 K
0.14	0.18	0.21	0.28	0.35

## Data Availability

Data is available from the authors upon request.
